# In Vivo Quasi-Elastic Light Scattering Eye Scanner Detects Molecular Aging in Humans and Mice

**DOI:** 10.1093/geroni/igab046.3406

**Published:** 2021-12-17

**Authors:** Douglas Parsons, Olga Minaeva, Srikant Sarangi, Danielle Ledoux, Juliet Moncaster, John Clark, David Hunter, Lee Goldstein

**Affiliations:** 1 Boston University School of Medicine, Boston, Massachusetts, United States; 2 Boston Children's Hospital, Boston, Massachusetts, United States; 3 University of Washington, Seattle, Washington, United States

## Abstract

The absence of clinical tools to evaluate individual variation in the pace of aging represents a major impediment to understanding aging and maximizing health throughout life. The lens is an ideal tissue for quantitative assessment of molecular aging in vivo. Long-lived proteins in lens fiber cells are expressed during fetal life, do not undergo turnover, accumulate molecular alterations throughout life, and are optically accessible in vivo. We used quasi-elastic light scattering (QLS) to measure age-dependent signals in lenses of both healthy human subjects and wild-type C57BL/6 mice. Age-dependent QLS signal changes detected in vivo in humans and mice recapitulated time-dependent changes in hydrodynamic radius, protein polydispersity, and supramolecular order of human lens proteins during long-term incubation (~1 year) and in response to sustained oxidation (~2.5 months) in vitro. Our findings demonstrate that QLS analysis of lens proteins provides a practical technique for noninvasive assessment of molecular aging in vivo.

